# Chemometric Optimization of SPE for the Determination of Multiclass Pesticides in Portable Water Samples by UHPLC-MS/MS

**DOI:** 10.1007/s00128-024-03873-2

**Published:** 2024-03-09

**Authors:** Garyfallia Drimaropoulou, Christophoros Christophoridis, Constantinos K. Zacharis, Konstantinos Fytianos

**Affiliations:** 1 Environmental Pollution Control Laboratory, Chemistry Department, Aristotle University of Thessaloniki, Thessaloniki, 54124 Greece; 2 Laboratory of Pharmaceutical Analysis, Department of Pharmacy, Aristotle University of Thessaloniki, Thessaloniki, 54124 Greece

**Keywords:** Pesticides, Solid phase extraction (SPE), Design of experiments, Central composite design (CCD), UHPLC-MS/MS

## Abstract

This study aimed on the development of a SPE-UHPLC-MS/MS method for the simultaneous determination of pesticide residues in drinking water samples. A chemometric approach was applied to optimize the efficiency of the SPE pretreatment procedure. This study involved (i) the application of a Full Factorial Design for the screening of the significant factors, (ii) the application of a Central Composite Design for the determination of the optimal conditions and (iii) the evaluation and validation of the significance of the statistically proposed models. Oasis HLB cartridges were used for the extraction. The optimum sample volume was 300 mL and the elution solvent 3 mL of the mixture of methanol:ethylacetate 70:30 v/v. The method was validated according to the international guidelines. Recoveries were ranged from 63 to 116% and the detection limits were between 0.1 and 1.5 pg mL^− 1^. The validated method could be used in routine analysis for pesticides screening.

## Introduction

Pesticides comprise a wide variety of chemical compounds widely used in agriculture to ensure the quality and quantity of agricultural products against to various treats from weeds, fungal diseases, insect pests etc. (Aktar et al. 2009). However, these compounds present various modes of toxicity to humans and other organisms, and they are responsible in many cases for the loss of biodiversity due to their ability to bio-accumulate in the food chain. Furthermore, they pose a constant and increasing threat to various environmental resources and especially to portable water ([CSL STYLE ERROR: reference with no printed form.]).

Several agencies have legislated various directives to ensure the quality of water for human consumption. For instance, European Commission established the *Council Directive 98/83/EC* by setting the maximum contamination levels for the summary of pesticides (insecticides, herbicides, fungicides, acaricides etc.) including their relevant metabolites, degradation and reaction products to be less than 0.5 µg L^− 1^ (Commission 1998). On the contrary, United States Environmental Protection Agency (US-EPA) is less restrictive since the maximum permitted concentration levels of some pesticides (atrazine, simazine) in portable water were established in the range of 3–4 mg L^− 1^ ([CSL STYLE ERROR: reference with no printed form.]).

Gas and liquid chromatography (GC, LC) are predominant separation techniques which are employed for the determination of multiclass pesticides in drinking water (Tankiewicz et al. 2010, 2013). Among them, liquid chromatography or ultra-high pressure liquid chromatography (UHPLC) especially coupled to mass spectrometric detection has proved to be a powerful analytical tool for the multi-residue analysis of pesticides in various matrixes offering high separation efficiency, low limits of detection and enhanced selectivity (Demoliner et al. 2010; Rubirola et al. 2017). Moreover, LC technique permits the determination of polar, non-volatile and thermal labile pesticides without the need of derivatization.

Due to the strict environmental legislation on pesticide residues and the requirement for ultra-trace analysis, a preconcentration / clean-up step is compulsory prior to analysis. Also, it is essential to minimize matrix effects in the ionization process and improve the accuracy of the method (Gosetti et al. 2010). Previous research on this topic involved the utilization of various sample preparation techniques comprising single drop-microextration (SDME) (Tian et al. 2014), dispersive liquid liquid microextraction (DLLME) (Zacharis et al. 2012b), vortex-assisted liquid liquid microextraction (Zacharis et al. 2012a), capsule phase microextraction (Ferracane et al. 2023; Manousi et al. 2023), etc. Apart from these techniques, solid phase extraction (SPE) in combination with LC–MS is considered to be the “gold standard” for this type of analysis offering versatility, rapidity, selectivity and sensitivity (Chen et al. 2023).

This work takes up the analytical challenge of performing trace-level detection and quantification of several classes of pesticides in drinking water applying multiple reaction monitoring (MRM) multiresidue analysis. The SPE conditions were optimized using a two-step chemometric approach, including an initial selection of the main SPE parameters affecting the measured response (recovery of compounds) and the subsequent application of an experimental design for the evaluation of the effect of these parameters and their interactions to the overall efficiency of the extraction procedure. Statistical design of experiments is an efficient statistical methodology to optimize a measured response based on analytical data, requiring reduced number of experiments, lower experimental cost, and various possibilities to evaluate interactions among variables.

The objectives of this study were: (a) to apply a chromatographic LC-MS/MS method for the simultaneous separation of 253 pesticides and their unambiguous qualitative and quantitative determination in water, (b) to develop, optimize and validate an SPE – LC- MS/MS method for the pretreatment / preconcentration of selected - representative multiclass pesticides (24) in water using experimental design.

## Experimental

### Reagents, Solutions and Materials

All pesticide standards were of quality grade appropriate for trace analysis and supplied from Sigma-Aldrich. Methanol (MeOH) and acetonitrile were of LC-MS grade and purchased from Merck (Darmstadt, Germany). Formic acid (LC-MS grade, 98%) and ethyl acetate were analytical grade and provided by Panreac (Spain). Water was produced by Direct-Q Millipore purification water system (Bedford, MA).

Individual standard stock solutions of the targeted analytes were prepared in methanol at concentration of 1000 mg L^− 1^. These solutions were stored at -20 ^o^C and protected from light. For the optimization of MS/MS parameters working standards solutions were prepared by serial dilution of standard pesticide solutions in MeOH/water 50/50% v/v. Calibration working standards solutions were prepared by dilution of the standard mixture pesticide solution with methanol to get a concentration range from 0.1 to 20 µg L^− 1^. For the SPE optimization a representative stock mixture of 24 multiclass pesticides (Table S1) was also prepared in MeOH at 100 mg L^− 1^. For method optimization a simulated groundwater sample was prepared by dissolving the appropriate salts in 1 L water to obtain 1 mg L^− 1^ K^+^, 37 mg L^− 1^ Na^+^, 73 mg L^− 1^ HCO_3_^−^, 63 mg L^− 1^ Ca^2+^, 16 mg L^− 1^ Mg ^2+^, 160 mg L^− 1^ Cl^−^ and 22 mg L^− 1^ SO_4_^2−^.

A 12-port Visiprep™ SPE vacuum manifold was purchased from Supelco (Bellefonte, U.S.A.). SPE extraction cartridges namely Oasis HLB (6 mL, 200 mg, 30 μm), Oasis HLB (6 mL, 500 mg, 60 μm) and Sep-Pak Vac C_18_ (3 mL, 500 mg, 55–105 μm) were purchased from Waters Co (Milford, MA, USA). Nylon filters (0.45 μm) for mobile phase filtration were obtained from Millipore Corporation (Bedford, MA, USA).

### *UHPLC – MS/MS* Conditions

All separations were carried out on Waters Acquity UHPLC-MS/MS TQD System (Waters, Manchester, UK) equipped with a thermostated autosampler and column oven. Triple quadruple mass spectrometer was equipped with an electrospray ionisation source (ESI) operated in positive ionization mode. All chromatographic separations were performed on a Waters Acquity UHPLC BEH C_18_ column (100 × 2.1 mm, 1.7 μm) (Milford, MA, USA) using a flow rate of 0.45 mL min ^− 1^. The mobile phases used were A: 0.1% v/v aqueous solution of formic acid/CH_3_OH, 98/2% v/v and B: 0.1% v/v methanolic solution of formic acid. The initial percentage of B was 5% and kept constant for 0.25 min and then was linearly increased to 100% in 7.75 min where it remained until 8.5 min of the analysis. Then the column was post-analysis equilibrated at initial mobile phase composition (5% B) for 1.5 min. The total separation time was 10 min. The column temperature was set constant at 40 ^o^C while the samples were thermostated at 5 ^o^C. The injection volume was 20 µL. Three replicates were performed for each sample.

The UHPLC system was coupled to a QqQ mass spectrometer equipped with an electrospray ionization (ESI) interface using the following operation conditions: source temperature 120 ^o^C; dessolvation temperature 450 ^o^C. Nitrogen (99.9995% pure) was produced by a high purity nitrogen generator, and it was used as a nebulizing, dessolvation and cone gas. Cone gas and desolation gas were set up to 50 and 1000 L h^− 1^. Dwell times were automatically selected in order to obtain sufficient points per peak. The instrument operation and data handled were controlled by MassLynx 4.1 software. Table S2 summarises the optimized MRM transitions, cone voltages and collision energies were obtained by direct infusion of each pesticide standard solution.

### Sample Collection and Preparation

All samples were collected in amber silanized bottles with Teflon faced caps (Fisher, UK). After filtration, samples were acidified with an aqueous solution of formic acid (10% v/v) to obtain a pH value of 3.0. Samples were stored in the dark at 4 ^o^C and extracted within 24 h.

Solid phase extraction cartridges Oasis HLB (6 mL / 200 mg, 30 μm) were utilized for the preconcentration of pesticides. The cartridges were initially activated using 5 mL methanol and then equilibrated with 5 mL of ultrapure water. A volume of 300 mL of water sample was applied to the SPE cartridge with a slight vacuum. The SPE cartridges were rinsed with 3 mL of ultrapure water and dried under vacuum. Then the pesticides were eluted using 3 mL of mixture methanol / ethyl acetate, 70/30% v/v followed by evaporation to dryness under a gentle stream of N_2_ in a water bath at 40 ^o^C. The dry residue was reconstituted by adding 1 mL of mixture CH_3_OH/ water, 50/50% v/v prior to LC-MS/MS analysis.

### Screening Design

Experimental design was conducted to decrease the number of experiments during the optimization of the SPE parameters and to assess the interaction between the variables (El-Osmani et al. 2014). The main factors influenced the SPE efficiency include sample volume, sample pH value, the volume and the elution solvent composition.

A two-level full factorial design was applied as a screening tool in order to evaluate the significant parameters. Table S3 demonstrates the studied variables, the coded ranges and the actual level values. The examined range of each factor was chosen based on the results collected from previous experimental data. The experimental matrix is composed of 16 runs and the experiments were performed in a random order (Table S4). All other chromatographic conditions were maintained constant.

### Central Composite Design (CCD)

A central composite design (CCD) through a quadratic model was developed between the dependent and the independent variables. The CCD is a widely used design typically for quadratic models fitting that combines a two-level factorial design with axial points (star points) and at least one point at the center of the experimental region to fit quadratic polynomials (Stalikas et al. 2009; Hibbert 2012). Center points are usually repeated to get a good estimate of experimental error (pure error). The value of “a” needed to ensure orthogonality and rotatability can be calculated from the following equation. The respective design consists of 11 experiments.

The evaluation of the statistical importance of the studied factors was carried out by using graphical tools including e.g. pareto chart, half-normal probability plot, normal plots, residual vs. predicted plots, analysis of variance (ANOVA), F-value, etc. were utilized to reveal the significance and predictability of the established model. In all cases, design generation and statistical analyses were performed by means of Design-Expert software 7.0.0 (Stat-Ease Inc., Minneapolis, MN, USA).

## Results and Discussion

### Preliminary Experiments

The first step of the preliminary experiments involves the selection of the most appropriate sorbent to retain all the compounds included in this study. Several SPE sorbents have been proposed in the literature for the preconcentration of the pesticides using polymeric-based phases (HySphere resin, Oasis HLB, Strata-X, Hamilton PRP-1, etc.) (Marín et al. 2006; Petrie et al. 2016; Rubirola et al. 2017) or silica-based phases (HySphere C_18_, Hypersil Gold etc.) (Viglino et al. 2008; Wode et al. 2012). In our study two polymeric-based namely Oasis HLB (6 mL / 200 mg, 30 μm), Oasis HLB (6 mL / 500 mg, 60 μm) and one silica-based SPE cartridge (Sep-Pak Vac C_18_ (3 mL / 500 mg, 55–105 μm) were investigated. The activation and preconcentration steps followed are described in Sect. 2.3 while the elution of the analytes was performed with 3 mL methanol. The experiments revealed that better overall recoveries for the majority of the pesticides were obtained with Oasis HLB sorbent and this type was adopted for the further study. Moreover, the sorbent mass (200 vs. 500 mg) had negligible effect on the recoveries of the analytes under the selected conditions and the mass of 200 mg was finally selected due to lower cost.

### Selection of Significant SPE Parameters – Screening Design

In order to minimize the experimental data obtained from the analysis of all analytes (total analytes 253) a representative mixture of 24 pesticides (3 pesticides from each group) was used for the optimization of the SPE conditions. These compounds exhibited different physicochemical characteristics and belong to different classes and pollution category (Table S1).

**Fig. 1:**
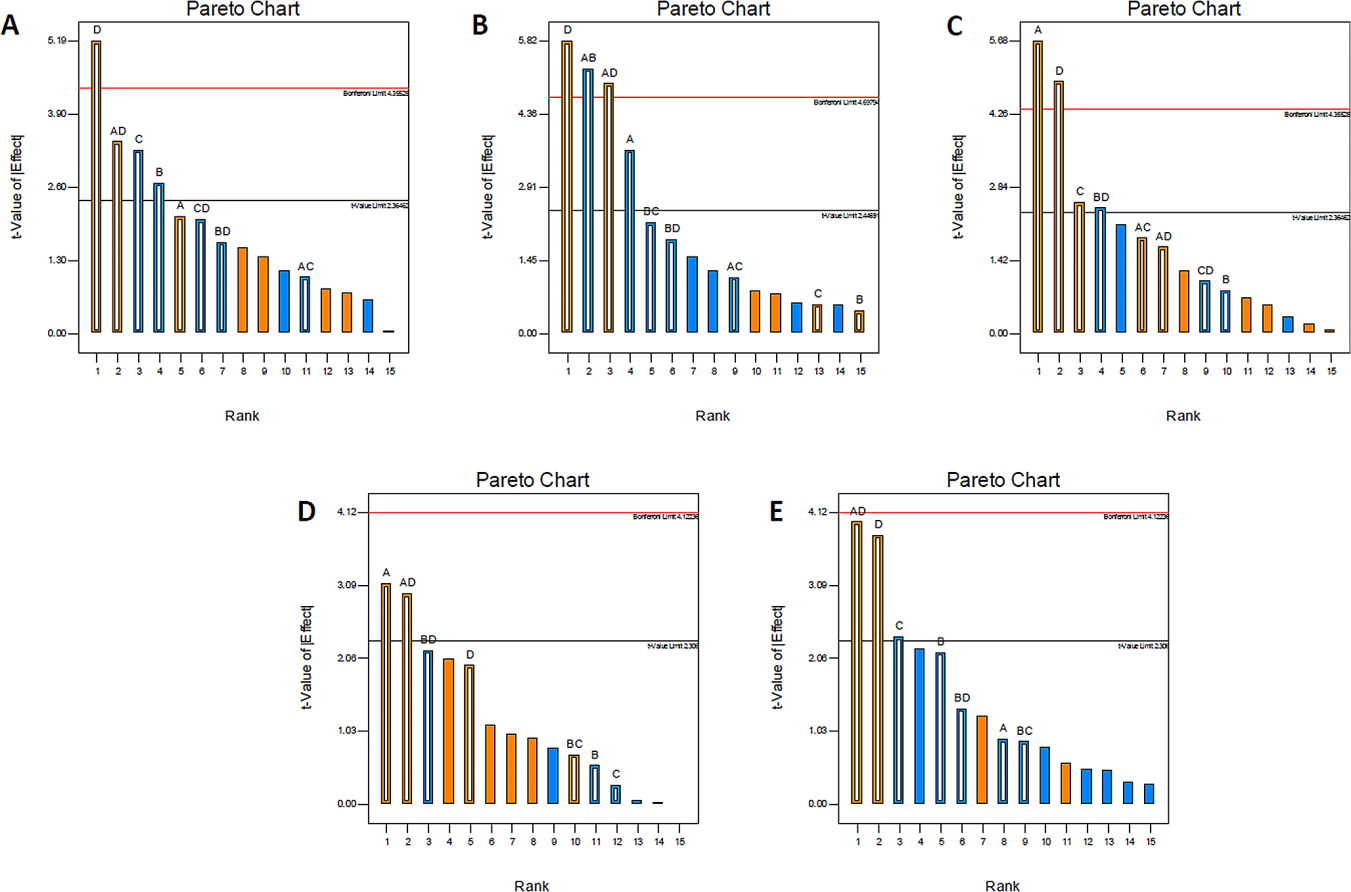
Pareto charts (sorted by order of importance) of the 4 studied factors (sample volume, sample pH, elution volume, elution solvent) for (**A**) pirimicarb, (**B**) imazalyl, (**C**) diazinon, (**D**) metolachlor, and (**E**) diuron

Four factors affecting the efficiency of SPE were initially screened using a full factorial experimental design (Table S4). Figure 1 illustrates the Pareto ranking plots of some representative pesticides which were derived from multivariate regression analysis. In these charts, effects above the “Bonferroni limit” were characterized as almost certainly significant while effects above the “*t*-value limit” were possibly significant (Stratigou et al. 2020; Mitsika et al. 2021). The bar length is indicative to the magnitude of their effects and the bar color designates the positive (orange) and the negative (blue) effect of the variable on the extraction recovery. In all cases F-values were higher than 4.0 indicating that the models are significant while the residuals follow a normal distribution in which the points follow a straight line.

As indicated in Fig. 1, the sample volume, the elution solvent and their interaction were statistically significant (*p* < 0.05) and their increase resulted in the increasing of the extraction recovery for the majority of the analytes. Although that the sample pH seems to have non-significant effect according to the Pareto charts, however the experiments indicated that the recoveries of polar pesticides (e.g. organophosphates, carbamates) were improved at lower pH values (data not shown). On this basis, the sample pH value of 3.0 was selected. Finally, the elution volume had no effect on the examined variable and the value of 3 mL was chosen for further studies.

In the case of pirimicarb, the Model F-value is 8.57 and there is only a 0.52% chance that this could occur due to noise. P-value 0.0052 (< 0.05) indicates that model D, C, B, AD terms are significant. Signal to noise ratio 10.371 is considered adequate since a ratio greater than 4 is desirable Fig. S1 depicts the diagnostic plots of this pesticide. In the normal probability plot, the residuals follow a normal distribution, in which case the points follow a straight line. Box Cox plot provides a guideline for selecting the correct power law transformation. The software did not recommend a specific transformation. The pareto chart shows that the ratio of elution solvent (D) is an important factor. In the case of diazinon, the Model F-value of 9.61 implies the model is significant. There is only a 0.37% chance that a “Model F-Value” this large could occur due to noise. Values of “Prob > F” indicates model terms A, C, D, BD are significant. The calculated signal to ratio 10.389 indicates an adequate signal. Figure S2 shows no need for specific transformation from Box Cox plot in the case of simazine. From Pareto chart, A and D variables are considered important.

In all cases, the results were studied as above. Figure 1C and E show the plots for other selected pesticides. As for chlorpyriphos, the recovery was low for all the set of the experiments performed. This suggested that a drastic change should be implied to the study concerning the elution solvent. Ethyl-acetate was introduced in the experimental design study to extract chlorpyriphos and other less polar analytes.

The results of this study showed that water sample acidification did not affect recovery significantly. Therefore, pH 7.0 was chosen as the working pH. Elution volume from the range 3–10 mL proved to be a likely significant factor. In order to improve the throughput, elution volume was set at 3 mL.

Sample volume and its interactions proved to be a significant factor for the SPE in most cases. Overall, the study of the models for the selected pesticides, led to the conclusion that further study of significant variables elution solvent composition (D) and sample volume (A) was necessary.

### Central Composite Design – parameter Relations, Effects and Optimization

Sample volume and elution solvent composition were selected as significant factors for response optimization. This design was used to optimize the above factors and therefore maximize the recovery of each analyte (Table S5). Experimental responses were fitted into the polynomial equation and response surface regression analysis was performed for each analyte. The significance and fit of the proposed model were evaluated based on statistical parameters. The *p* value was less than 0.05, so the proposed models were statistically important. Lack of fit was more than 0.05, so the experimental data fits the models.

Numerical optimization was performed, aiming at recoveries of 100% for each selected pesticide. The scope of optimization was to calculate a common set of conditions that will meet the goal, based on a common optimization function derived by the mathematical models obtained for each pesticide.

The optimum set of conditions was selected in order to obtain a time saving preconcentration SPE step requiring less sample volume and quick evaporation. Sample volume of 300 mL and elution solvent composition 70:30 v/v MeOH: ethyl acetate were found to be the best experimental conditions for the quantitative extraction of solid phase extraction of the selected pesticides.

### Method Validation

**Fig. 2:**
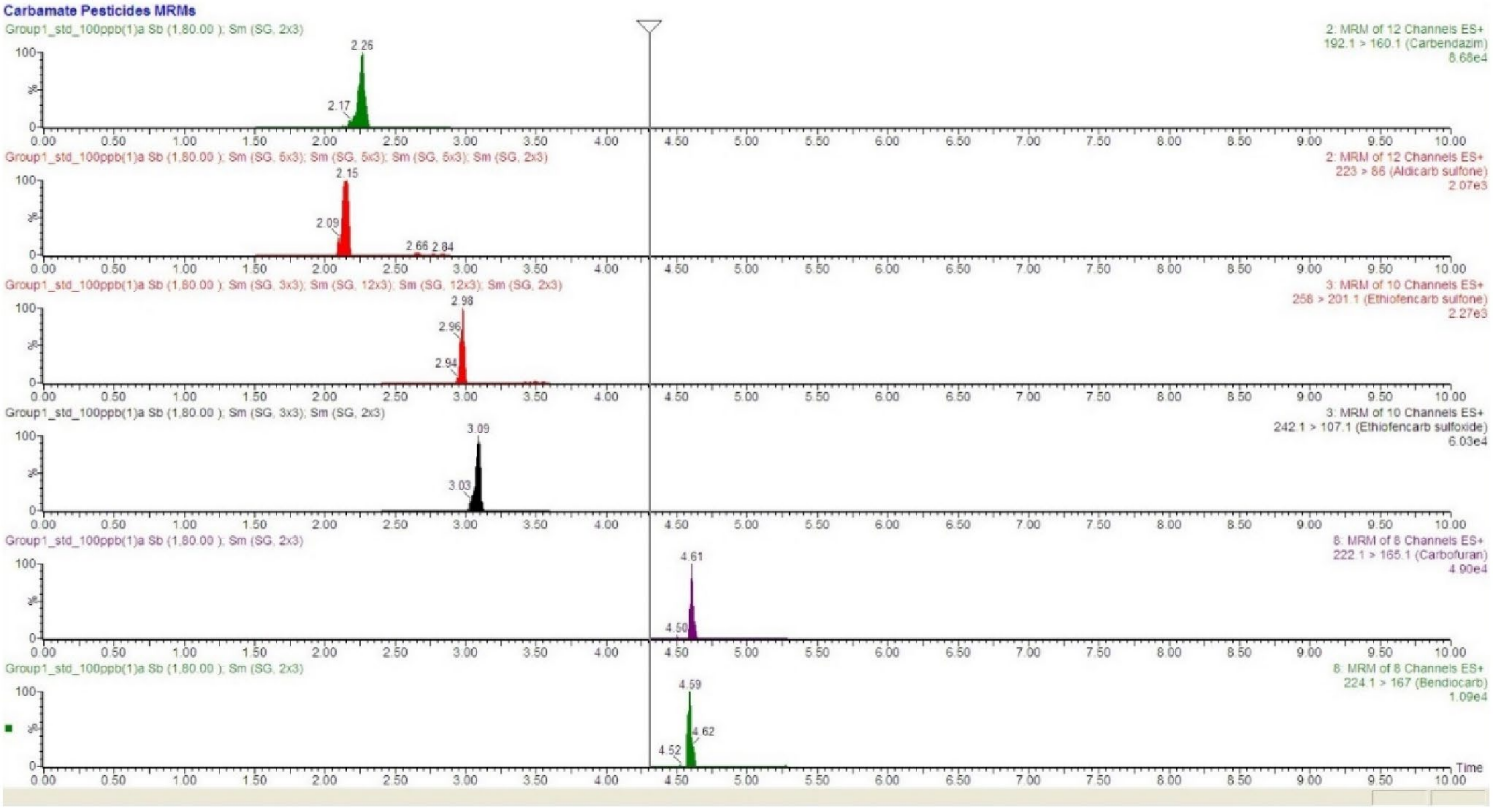
Representative MRM chromatograms for the analysis of selected carbamate pesticides using the proposed SPE-UHPLC-MS/MS approach

The SPE-UHPLC-MS/MS method was validated using simulated drinking water samples in terms of linearity, accuracy and precision, limit of detection (LOD) and quantitation (LOQ) (Sidiropoulou et al. 2022). The correlation coefficients of calibration curves were higher than 0.99 in all cases indicating acceptable linearity in the studied concentration range of LOQ – 80 ng mL^− 1^. The limits of detection (LODs) (based on S/*N* = 3 criterion) ranged between 0.1 and 1.5 pg mL^− 1^ for all pesticides studied. The intra-day and inter-day precision and accuracy data of the developed SPE-UHPLC-MS/MS method are given in Table 1. The intra-day and inter-day accuracy and precision intra-day were evaluated at three concentration levels of 5, 25 & 80 ng mL^− 1^. For all levels, the intra-day accuracy (% relative recovery) was varied from 63 to 116% while the precision (RSD %) (*n* = 3) was lower than 25%, respectively. The inte-day accuracy ranged between 74 and 115% and with %RSD lower tha 30%. Although non-deuterated ISTDs are employed for quantification purposes the above results are acceptable. Representative MRM chromatograms of some pesticides are shown in Fig. 2 and Figs. S3-S6. The applicability of the verified by the analysis of portable sample spiked with the studied analytes.

**Table 1:** SPE-UHPLC-MS/MS validation data for the selected pesticides

				% Relative recovery (RSD)					
				Intra-day (*n* = 3)	Inter-day (*n* = 3)	Intra-day (*n* = 3)	Inter-day (*n* = 3)	Intra-day (*n* = 3)	Inter-day (*n* = 3)
Analyte	LOD (pg mL^− 1^)	LOQ (pg mL^− 1^)	R	5 ng mL^− 1^	5 ng mL^− 1^	25 ng mL^− 1^	25 ng mL^− 1^	80 ng mL^− 1^	80 ng mL^− 1^
Pirimicarb	0.2	0.5	0.998	101(6)	97 (25)	102 (19)	110 (30)	97 (9)	99 (23)
Carbendazim	0.6	0.9	0.994	87(8)	94(15)	97(8)	98(10)	96(17)	103(13)
Esprocarb	0.2	0.5	0.996	76(15)	102 (14)	103 (5)	115 (10)	97 (16)	99 (14)
Carbaryl	0.3	0.5	0.990	110(12)	96 (10)	96 (15)	99 (18)	105 (10)	111(16)
Atrazine	0.6	0.9	0.996	99(15)	95 (16)	99 (8)	100 (8)	98 (10)	105 (12)
Simazine	0.2	0.5	0.998	85(8)	99(22)	98 (15)	98 (13)	102 (10)	102 (20)
Prometryn	0.9	1.2	0.996	97(13)	74 (20)	73 (20)	77 (19)	71 (10)	79 (12)
Imazalyl	0.1	0.3	0.994	95(19)	79 (15)	82 (19)	85 (19)	85 (25)	98 (27)
Chlorfenvinphos	0.9	1.4	0.992	87(9)	110 (27)	98 (9)	100 (12)	97 (10)	97 (10)
Chlorpyriphos	1.5	3.5	0.992	66(13)	74 (25)	71 (12)	71 (17)	70 (8)	79 (30)
Diazinon	0.9	1.6	0.996	89(7)	88 (21)	86 (12)	90 (19)	86 (14)	95 (14)
Pyrazophos	0.6	1.2	0.996	65(6)	100 (19)	99 (10)	99 (10)	98 (15)	99 (18)
Dichlorvos	0.2	0.5	0.996	97(7)	96 (15)	97 (10)	105 (10)	98 (10)	98 (10)
Metolachlor	0.8	1.2	0.996	66(16)	92(21)	92 (5)	107 (15)	95 (15)	100 (8)
Metalaxyl	0.6	0.9	0.998	69(5)	96 (17)	95 (9)	92 (9)	95 (10)	100 (10)
Picolinafen	0.1	0.4	0.998	65(4)	100 (17)	100 (15)	100 (11)	98 (9)	98 (9)
Pyrimethalin	0.9	1.4	0.998	70(13)	106(21)	98 (14)	99 (25)	100 (16)	108 (16)
Isoproturon	1.0	2.5	0.996	70(12)	99 (27)	95 (22)	105 (28)	93 (17)	98 (27)
Chlortoluron	1.3	3.0	0.990	86(10)	90 (13)	95 (13)	98 (19)	96 (8)	99 (10)
Linuron	0.5	0.9	0.994	106(8)	96 (15)	105 (19)	106 (22)	106 (5)	110 (28)
Monuron	0.9	2.5	0.992	63(12)	110 (12)	100 (10)	101 (13)	116 (20)	119 (25)
Diuron	0.8	2.5	0.992	99(9)	112 (9)	100 (10)	108 (10)	112 (9)	109 (19)
Pyridaben	0.1	0.3	0.992	66(7)	108 (19)	105 (18)	110 (20)	102 (20)	115 (11)
Pendimethaline	0.2	0.5	0.992	89(12)	103 (18)	98 (19)	99 (17)	95 (23)	98 (25)

## Conclusion

This research studied the development of a SPE-UHPLC-MS/MS method for the simultaneous determination of pesticide residues in drinking water samples. Experimental design was applied for the optimisation of SPE procedure. A two-level full factorial design was used with regard to factors such as sample pH, sample volume, elution solvent composition, and volume. These variables were evaluated statistically, indicating the main effects. The significant variables were optimised using central composite design (CCD). The developed analytical method was fast, cost-effective and green sample preparation tool offering selectivity with relatively fast extraction kinetics avoiding additional pre-treatment steps. Compared to other extraction-based sample preparation techniques, SPE exhibited several advantages including – among others – relative high extraction recoveries and robustness. The developed analytical scheme could be established as a suitable routine procedure for screening ultra-trace levels of pesticides in drinking water.
